# 第三代EGFR-TKIs耐药之初探

**DOI:** 10.3779/j.issn.1009-3419.2018.02.02

**Published:** 2018-02-20

**Authors:** 莲芳 倪, 立功 聂

**Affiliations:** 1 100034 北京, 北京大学第一医院老年内科 Department of Geriatric Medicine, Peking University First Hospital, 100034 Beijing, China; 2 100034 北京, 北京大学第一医院呼吸和危重症科 Department of Respiratory and Critical Care Medicine, Peking University First Hospital, 100034 Beijing, China

**Keywords:** EGFR受体, 肺肿瘤, 第三代EGFR-TKIs, 耐药, Epidermal growth factor receptor, Lung neoplasms, Third-generation tyrosine kinase inhibitors, Resistance

## Abstract

表皮生长因子受体酪氨酸激酶抑制剂（epidermal growth factor receptor-tyrosine kinase inhibitors, EGFRTKIs）靶向治疗已成为EGFR基因突变晚期非小细胞肺癌（non-small cell lung cancer, NSCLC）患者的一线治疗方法。第三代EGFR-TKIs用于一、二代TKIs耐药EGFR T790M突变NSCLC的治疗，给晚期肺癌患者带来更多的生存获益。然而，第三代EGFR-TKIs应用一段时间后不可避免地会出现耐药。肿瘤的异质性决定了耐药机制的多样性，第三代EGFR-TKIs的耐药包括依赖EGFR通路（新发突变、T790M减少或消失和EGFR基因扩增等）和不依赖EGFR通路（旁路途径的激活和细胞表型的转变）两大类，现就此问题进行简单的综述。

## Abstract (en)

Targeted therapy of epidermal growth factor receptor tyrosine kinase inhibitor (EGFR-TKIs) has been the standard modality as first-line treatment of advanced *EGFR*-mutated non-small cell lung cancer (NSCLC). The thirdgeneration EGFR-TKIs has been approved to overcome the *EGFR* T790M mutation in patients resistant to the first-or secondgeneration TKIs, which brings more survival benefits for patients with advanced NSCLC. Unfortunately, acquired resistance inevitably develops after application of approximately 10 months. Heterogeneities of the tumor determines the diversity of resistance. Mechanisms of resistance to the third-generation TKIs includs EGFR-dependent pathway (such as new *EGFR* mutations, T790M reduction/disappearance and *EGFR* amplification, *etc*.) and EGFR-independent pathway (such as bypass pathway activation and histological transformation, *etc*.). In this paper, we reviewed principle mechanisms of acquired resistance to third-generation EGFR-TKIs.

肺癌是全球威胁人类生命健康最大的恶性肿瘤，在我国也是如此，其发病率和病死率占所有恶性肿瘤的18.74%和25.24%，并有逐年上升的趋势^[1]^。但是肺癌预后并不尽如人意，5年生存率仅17%^[2]^，主要是因为大多数肺癌在诊断时已经是局部晚期或晚期，失去了手术治疗的机会。当前在内科治疗方面，包括化疗、靶向治疗和免疫治疗均取得了长足的进步，尤其是在表皮生长因子受体（epidermal growth factor receptor, *EGFR*）敏感突变的非小细胞肺癌（non-small cell lung cancer, NSCLC）更是如此，第一代和第二代表皮生长因子受体酪氨酸激酶抑制剂（EGFR tyrosine kinase inhibitors, EGFR-TKIs）已成为*EGFR*突变阳性晚期NSCLC患者的标准一线治疗。目前我们可以在第一代EGFR-TKIs出现耐药后进行检测，选择应用第三代EGFR-TKIs。新近的FLAURA研究显示^[3]^，第三代EGFR-TKI奥西替尼一线治疗*EGFR*突变阳性的局部晚期或转移性NSCLC，与标准治疗相比（吉非替尼或厄洛替尼），可进一步延长患者的中位无进展生存（median progression-free survival, mPFS）（18.9个月*vs* 10.2个月），降低疾病进展和死亡风险达54%（HR=0.46; 95%CI: 0.37-0.57; *P* < 0.000, 1），给晚期肺癌患者带来巨大的生存获益。然而，第三代EGFR-TKIs应用一段时间后不可避免地会出现耐药，现就此问题进行简单的综述。

EGFR-TKIs的耐药机制包括了固有耐药和获得性耐药两大类。固有耐药目前原因不明，但解释了敏感突变对各代EGFR-TKIs的客观有效率并不能达到100%的原因。获得性耐药是肿瘤细胞在接触药物后通过各种变异规避药物的影响，反映了肿瘤异质性和肿瘤细胞的进化，也是耐药机制及耐药对策研究的重点。根据是否依赖EGFR通路，第三代EGFR-TKIs的耐药机制可以分为依赖EGFR和不依赖EGFR通路两大类，前者包括新发突变、T790M减少或消失和*EGFR*基因扩增等，后者包括旁路途径的激活和细胞表型的转变等，下文具体阐述。

## 1 依赖EGFR通路

### 1.1 新发*EGFR*突变

*EGFR*-C797S突变是导致第三代TKI耐药最常见的继发突变。C797S是EGFR 20号外显子797位点上丝氨酸取代了半胱氨酸的错义突变，位于EGFR的酪氨酸激酶区，C797S的突变使得奥西替尼（AZD9291）无法在ATP结合域内继续形成共价键，从而失去抑制EGFR激活的效果，导致耐药的发生。该突变最早发现于AZD9291治疗的Ⅰ期AURA试验患者的血浆游离DNA（cell-free plasma DNA, cfDNA）中（15例患者中有6例，40%）^[4]^。液滴数字PCR技术检测发现，T790M突变患者AZD9291治疗耐药后会出现3种分子亚型，在检测的15例患者中，6例新发C797S突变且仍存在*EGFR*敏感突变和T790M突变（40%），5例维持T790M突变但无C797S突变（33%），4例T790M突变消失也无C797S突变但仍存在*EGFR*敏感突变（27%）。随后的研究发现^[5]^，C797S与T790M突变是否在同一等位基因具有重要的生物学意义，会影响后续TKIs的治疗效果。*EGFR* C797S突变与T790M突变主要呈顺式构型（位于同一染色体上），占85%，对目前获批的EGFR-TKIs单药或联合治疗均耐药。约10%患者为C797S/T790M反式构型（位于不同染色体上），肿瘤细胞对第三代EGFR-TKIs耐药，但对第一代和第三代TKIs联合治疗敏感。研究还发现小部分患者仅有C797S突变，未合并T790M突变，如果C797S在T790M野生型细胞中发生，则会导致第三代TKIs耐药，而保留对第一代TKIs的敏感性。我国吴一龙教授团队报道了第一代和第三代EGFR-TKI联合治疗*EGFR* T790M和C797S反式突变有效的临床病例，而且首次发现经过厄洛替尼和奥西替尼联合治疗后*EGFR* T790M和C797S从反式向顺式演变并介导耐药，提示在靶向治疗的每个阶段进行基因检测是非常有必要的^[6]^。C797S突变在应用不同第三代EGFR-TKIs发生率也有明显差异，应用奥西替尼发生率较高，约40%；应用rociletinib发生率较低，约2%^[7]^。Sequist等^[8]^还报道9例rociletinib耐药后改为奥西替尼治疗的患者中，3例PR，4例SD，2例PD，中位PFS 208 d。其中6例rociletinib耐药后直接进行奥西替尼治疗的患者均达到了PR或SD。3例rociletinib治疗过程中CNS进展的患者接受奥西替尼治疗后CNS病灶控制良好。提示rociletinib耐药可能是由于靶向抑制不完全，奥西替尼可逆转这种耐药，包括CNS进展。C797S突变有时呈多克隆，常与其他靶向耐药机制共存，这提示*EGFR*突变患者耐药后的肿瘤呈异质性，ctDNA的检测可能会比组织活检更能反映疾病的全貌^[9]^。

除了C797S，陆续有新的耐药点突变被证实，如L798I、L692V、L718Q、E709K、G724S、G796R、G796D、G796S、L792F/H等^[7, 10-13]^。体外实验^[14]^表明，*EGFR* L858R/T790M/C797S三突变的细胞对变构抑制剂第四代TKI EAI045联合西妥昔单抗敏感。EAI045单药抑制作用弱，联合西妥昔单抗在鼠肺癌模型中可使肿瘤缓解^[15]^。而体外和在体试验^[16]^均显示，间变性淋巴瘤激酶（anaplastic lymphoma kinase, ALK）抑制剂brigatinib联合抗EGFR抗体对C797S/T790M/del19三突变的细胞敏感。这可能是3代TKIs耐药后我们的研究方向。

### 1.2 T790M减少或消失

T790M突变减少或消失可能是第三代TKIs的选择性压力的结果，使T790M突变阴性克隆成为主导，也反映了肿瘤的异质性。Thress等^[4]^报道的奥西替尼治疗耐药的15例患者中，4例出现T790M突变消失。Piotrowska等^[17]^报道的rociletinib治疗后耐药的12例患者中6例出现T790M突变消失，其中2例转变为小细胞肺癌（small cell lung cancer, SCLC）。T790M的突变负荷可以预测第三代TKIs治疗的效果，突变负荷越大，效果越好。相反，T790M消失的患者奥西替尼治疗的效果更差^[18]^。

### 1.3 *EGFR*基因扩增

*EGFR*基因扩增更多见于rociletinib治疗的患者，可以合并存在T790M突变。Piotrowska等^[17]^报道的rociletinib耐药T790M突变存在的7例患者中，3例存在*EGFR*基因扩增，Chabon等^[7]^报道rociletinib耐药患者中有9%出现*EGFR*基因拷贝数的变化。细胞试验中，第三代TKI WZ4002耐药的细胞株也可以观察到*EGFR*基因扩增^[5]^。*EGFR*基因扩增可能会导致TKIs类药物浓度相对不足，导致耐药。

## 2 不依赖于EGFR通路

### 2.1 *MET*和*HER2*扩增

*MET*和*HER2*扩增作为重要的旁路激活途径，在一代及三代TKI药物耐药机制中都具有重要地位。MET属于肝细胞生长因子受体，具有酪氨酸激酶活性，参与细胞信息传导、细胞骨架重排的调控，是细胞增殖、分化和运动的重要因素。离体和在体实验^[19]^发现，MET与EGFR可以形成二聚体，此种二聚体依赖于*EGFR*的突变形式，在L858R+T790M中出现，加入MET抑制剂后能够抑制肿瘤细胞的增殖。Planchard等^[20]^最早报道了AZD9291耐药的患者中存在*MET*和*HER2*扩增，并伴T790M突变的消失。在第三代EGFR-TKI耐药中，*MET*扩增比较常见，rociletinib耐药的患者中26%存在*MET*扩增^[7]^，MET抑制剂克唑替尼单用或与三代TKIs联和用药可以很好地抑制三代TKIs耐药肿瘤细胞的生长。在奥西替尼耐药的患者中也可以检测到*MET*扩增，克唑替尼单药治疗有效^[21]^。Martinez-Marti等^[22]^在奥西替尼耐药患者脑转移病灶中也检测到了*MET*扩增，c-Met抑制剂capmatinib联合ErbB-1/2/4抑制剂阿法替尼能完全抑制脑转移细胞原位注射小鼠肿瘤的生长。Planchard^[20]^及Oxnard等^[23]^对AZD9291耐药患者的基因检测分析中发现了HER2的扩增。在rociletinib治疗耐药的患者中也观察到类似的HER2的扩增^[7]^。体外研究中^[24]^，曲妥珠单抗的药物偶联物T-DM1可以延缓或克服奥西替尼的耐药。HER2和MET扩增可以在应用第三代EGFR-TKI之前就存在，也可以在应用第三代EGFR-TKI之后出现，伴或不伴T790M突变消失。应用第三代EGFR-TKI之前就存在MET扩增的患者对第三代EGFR-TKI反应更差^[25]^。

### 2.2 *PIK3CA*基因突变

通过PI3K/AKT/mTOR途径激活的PI3K脂质激酶家族亚基*PIK3CA*是肺腺癌的致癌驱动基因之一，常与*HER2*、*MET*及*EGFR*等其他突变合并存在，存在*PIK3CA*突变的肺癌患者中位生存时间更短^[26, 27]^。Chabon等^[7]^报道了rociletinib耐药患者的*PIK3CA*基因突变，发生率约12%。在AZD9291治疗的患者中也有*PIK3CA*基因突变的报道^[23]^。

### 2.3 同源丢失性磷酸酶-张力蛋白（phosphatase and tension homology deletechromosometen, *PTEN*）基因缺失

*PTEN*是一个具有双特异性磷酸酶活性的抑癌基因，通过抑制PI3K/Akt途径的活化而抑制细胞增殖，与人类许多癌症有关，也是第一代TKI耐药的机制之一^[28]^。体外研究^[29]^发现，活化核糖核酸（RNAa）可以通过上调PTEN蛋白表达而逆转吉非替尼的耐药。Kim等^[30]^报道一个*EGFR* T790M突变的病例，在奥西替尼治疗前即存在*PTEN*缺失突变，奥西替尼治疗后PTEN缺失的肿瘤比例和EGF mRNA水平增加，推测可能参与了局部的奥西替尼耐药。

### 2.4 RAS-MAPK通路活化

对*EGFR*突变细胞系的耐药机制临床前研究显示^[31]^，AZD9291获得性耐药与RAS信号通路激活有关，包括*NRAS*基因E63K突变，野生型NRAS及*KRAS*扩增。在奥西替尼和rociletinib治疗的患者中均发现了不同位点的*KRAS*活化突变，部分可与PIK3CA和MET等途径共同存在^[7, 25]^。Kim等^[30]^在奥西替尼治疗的患者中检测到了*MAPK1*基因的扩增，而且研究^[25, 31]^发现，MEK抑制剂如selumetinib和trametinib联合第三代TKIs可以克服MAKP信号途径的耐药，目前Ⅰ期临床研究正在进行。

### 2.5 成纤维细胞生长因子2-成纤维细胞生长因子受体1（fibroblast growth factor 2-fibroblast growth factor receptor 1, FGF2-FGFR1）

Kim等^[30]^报道了FGF2-FGFR1自分泌环介导的奥西替尼耐药，研究发现肿瘤有局灶性*FGFR1*基因扩增和与基线相比20倍的FGF2 mRNA增长，且伴有*EGFR* T790M突变的消失。

### 2.6 胰岛素样生长因子-1受体（Insulin-like growth factor-1 receptor, IGF1R）通路

IGF1R主要通过下游PI3K/Akt信号通路参与调节细胞生长、分化、凋亡、转化和其他重要的生理过程，IGF1R导致EGFR-TKI继发耐药也主要是通过PI3K-AKT途径实现的。临床前研究显示^[32]^，在wz4002耐药的两个细胞系中出现了IGF1R的异常激活伴胰岛素样生长因子结合蛋白-3的损失（IGFBP3），在体外实验和种植瘤模型中，抑制IGF1R活性的小分子或单克隆抗体的可以恢复wz4002敏感性，提示有可能应用IGF1R抑制剂联合EGFR-TKIs治疗来克服耐药。

## 3 细胞表型的转变

### 3.1 肿瘤细胞类型转变

在第一代和第三代EGFR-TKIs治疗后都能观察到NSCLC的SCLC转化，其在第一代EGFR-TKI的耐药机制中占11%^[33]^。Lee等^[34]^研究发现，*Rb1*和*p53*基因失活的*EGFR*突变腺癌更易发生SCLC转化。Piotrowska等^[17]^最先报道了2例SCLC转化导致的rociletinib耐药，随后Kim^[30]^及Ham等^[35]^报道了SCLC转化导致的奥西替尼耐药。Ham等^[35]^报道的AZD9291 Ⅰ期临床研究的2例耐药患者接受依托泊苷联合卡铂化疗后肿瘤均明显缩小，提示小细胞转化可能为第三代EGFR-TKIs耐药机制。我国也有类似的病例报道^[36]^。与第一代TKIs耐药相似，肿瘤保持典型的*EGFR*驱动突变，表明SCLC由*EGFR*突变克隆进化而来，但T790M突变消失，抑癌基因*RB1*的基因组缺失或突变。AURA研究中奥西替尼治疗进展的22例患者中，除了检测到2例SCLC外，还发现1例鳞癌转化^[18]^。因此，对于TKIs耐药的患者，除了液体活检检测相关突变基因之外，还需要同时强调组织活检的重要性，来明确有无组织学类型的转变^[37]^。

### 3.2 上皮-间充质转化（epithelial-mesenchymal transition, EMT）

Walter等^[38]^报道，CO1686获得性耐药与上皮-间质转化及对AKT抑制剂的敏感性增加有关。Sakuma等^[39]^的体外研究发现，脯氨酰异构酶Pin1能促进*EGFR*突变型肺腺癌细胞的上皮-间质表型转化。

## 4 其他

离体和在体研究均发现^[40]^，Src通路激活与EGFR野生型等位基因扩增也会导致第三代TKIs的耐药（rociletinib，奥西替尼）。BIM，也叫BCL-2蛋白11，是一种促凋亡分子，属于bcl-2家族。目前研究认为BIM蛋白缺失多态性同样参与了第三代TKI s奥西替尼的耐药，而组蛋白去乙酰化酶3抑制剂可以通过上调BIM的表达来克服这种耐药^[41]^。在奥西替尼耐药患者的胸水细胞中，检测到了*BRAF* V600E基因突变，*BRAF* V600E抑制剂可以抑制肿瘤细胞生长，联合应用奥西替尼效果更好^[42]^。

综上，第三代EGFR-TKIs的耐药机制与第一代类似，但是更为复杂，往往多种机制并存，rociletinib耐药时46%患者存在一种以上的耐药机制^[7]^。出现耐药后需要更为准确的血液、组织学检测，寻找可能的耐药机制，为临床下一步治疗提供线索。
